# Spatio-temporal evolution of female lung cancer mortality in a region of Spain, is it worth taking migration into account?

**DOI:** 10.1186/1471-2407-8-35

**Published:** 2008-01-31

**Authors:** Oscar Zurriaga, Hermelinda Vanaclocha, Miguel A Martinez-Beneito, Paloma Botella-Rocamora

**Affiliations:** 1 Dirección General de Salud Pública, Conselleria de Sanidad, Valencia, Spain; 2 Departamento de Ciencias Físicas, Matemáticas y de la Computación, Universidad CEU-Cardenal Herrera, Moncada, Spain

## Abstract

**Background:**

The Comunitat Valenciana (CV) is a tourist region on the Mediterranean coast of Spain with a high rate of retirement migration. Lung cancer in women is the cancer mortality cause that has increased most in the CV during the period 1991 to 2000. Moreover, the geographical distribution of risk from this cause in the CV has been previously described and a non-homogenous pattern was determined. The present paper studies the spatio-temporal distribution of lung cancer mortality for women in the CV during the period 1987–2004, in order to gain some insight into the factors, such as migration, that have had an influence on these changes.

**Methods:**

A novel methodology, consisting of a Bayesian hierarchical model, is used in this paper. Such a model allows the handling of data with a very high disaggregation, while at the same time taking advantage of its spatial and temporal structure.

**Results:**

The spatio-temporal pattern which was found points to geographical differences in the time trends of risk. In fact, the southern coastal side of the CV has had a higher increase in risk, coinciding with the settlement of a large foreign community in that area, mainly comprised of elderly people from the European Union.

**Conclusion:**

Migration has frequently been ignored as a risk factor in the description of the geographical risk of lung cancer and it is suggested that this factor should be considered, especially in tourist regions. The temporal component in disease mapping provides a more accurate depiction of risk factors acting on the population.

## Background

Lung cancer is the most common cause of death from cancer among males in the European Union (EU), and the second most common among females [1]. Lung cancer mortality in women has increased sharply in almost all EU countries in recent decades [2] with an annual growth of 1.7 percent [3], and Spain is not an exception to this trend [4]. However, although the Spanish female adjusted mortality rate for lung cancer is relatively low, the observed mortality increase in the last decade of the twentieth century for women between ages 35 and 64 is the highest in Europe [5], and an increase in incidence has been noted as well [6].

Lung cancer is a long latency disease [7] mainly reflecting past smoking habits. In fact tobacco smoking is well-established as the main cause of lung cancer and about 90 percent of cases are thought to be tobacco related [8]. In Spain, a lung cancer risk increase related to smoking patterns has been described [9] for female generations born after 1940. However, smoking prevalence in women is not the same in every European country. In northern Europe, more women smoke than in the southern countries and Denmark, for example, is one of the countries in the world with the highest prevalence [10]. For populations in which smoking prevalence is relatively low (in Spain, for example) increases in the prevalence of daily cigarette smoking have been observed [11]. Meanwhile, in northern European countries, smoking rates for both males and females have begun to decrease [12]. As lung cancer mortality patterns reflect past smoking habits, substantial increases in female lung cancer mortality can be expected in most European countries except in those that have a decreasing smoking prevalence among women [13].

The Comunitat Valenciana (CV) is a Spanish autonomous region (4,692,449 inhabitants in 2005) on the Mediterranean coast with a very strong tourist sector and a high rate of international retirement migration. In the CV, the female lung cancer annual standardized mortality rate increased by 53.4 percent from 1991 to 2000, and a non-homogeneous geographical pattern in mortality has also been described during that decade [14]. At the moment, the impact of immigration on this increase has neither been described nor quantified, immigration being either for economic (from low-income countries) or retirement reasons.

On the other hand, the exploration of geographical patterns on a small-area level can provide clues about the risk factors underlying the observed mortality rates. For example, in the USA female lung cancer mortality maps are consistent with a cohort effect of decreasing exposure by age [15], but the concordance between geographical patterns of smoking prevalence and lung cancer is less pronounced for females than for males [16]. Therefore, our aim is to study the spatio-temporal evolution of lung cancer mortality in women at a small-area level in the CV. With this in mind, we will use a new approximation for spatio-temporal analysis in order to verify whether the former increase has been experienced to the same degree in the whole CV or if there are some regions with larger increases than others. If this is the case, the regions with a higher increase in values would provide important insights into what factors can have had a greater influence on the evolution of risk. Thus, the impact of migration could be made evident if higher increases could be related to those regions with more immigration.

## Methods

Mortality and population data were available for the whole CV for the period 1987 to 2004 with a municipal and yearly disaggregation. There were about 2.3 million women in the CV in 2004 distributed across 540 municipalities, and the median number of women per municipality for that year was 683. This high disaggregation level in data, together with a crude female mortality rate from lung cancer in the CV during the period of study of 7.81 deaths for every 100.000 women, requires the use of a statistical model that takes advantage of the spatial and temporal dependence on data, in order to reliably estimate the geographical distribution of risk and its temporal evolution in every municipality.

Mortality data were provided by the mortality registry of the CV, corresponding to all the deaths registered with the 9^th ^revision ICD code of 162 [17] from 1987 to 1997 and the 10^th ^revision ICD codes C33–C34 [18] from 1998 to 2004. Population data was obtained from the Spanish National Statistics Institute for the years 1991, 1996 and for the whole period 1998–2004. For those years in which population figures were not provided, they were estimated by geometric interpolation. With all this information, expected death counts for every municipality and year were calculated under the hypothesis that risk remained constant throughout the whole period and region under study.

Statistical modelling for observed death counts was made by means of a Bayesian mixed effects Poisson regression including one random effect for every municipality and year. This effect has spatial structure, in such a way that neighbouring regions will share similar risk values. Nevertheless, the shortage of data resulting from the available disaggregation level means that a consideration of risks for consecutive years, in every municipality, as temporally independent quantities is not recommended. Moreover, it seems reasonable to think that risks for every municipality were similar in consecutive years, and so it would be appropriate to consider this fact in order to define the dependence structure in the data. The model used for our analysis imposes an order-one autoregressive temporal dependence structure for every municipality, in order to share information on risk estimates through time in a similar way to the one used to share information in space. More information on this modelling can be found in Martinez-Beneito et al. [19], where further technical details are given. The present study supposes the first application of the methodology already described in the mentioned paper to a real problem. The model used for our analysis does not include a heterogeneous term for every municipality and year as is usually done in spatial studies; this is because we have checked that such a model provides a worse fit in terms of the DIC model selection criterion [20]. Moreover, in Martinez-Beneito et al. [19], it is also shown that the model with heterogeneous terms would provide a worse fit unless the number of observed deaths was high enough to support it and that does not seem to be our case.

The statistical software used to make the inference on this model was WinBUGS 1.4.1 [21]. The R2WinBUGS library of the R [22] statistical package has also been used as an interface to run the model and perform some convergence tests. The WinBUGS code corresponding to the model is available as supplementary material [see Additional file 1].

In order to get all the posterior distributions that we are interested in, three chains have been run with 10,000 iterations in each one. The first 5,000 iterations of every chain were discarded in order to ensure that convergence had been reached at the moment the simulations were saved. From that point on, samples for every 15th iteration have been stored in order to avoid computational storage problems. Thus the final sample size for every parameter adds up to 1,000 values. Once the iteration process was complete, the Brooks-Gelman-Rubin statistic [23] and the effective number of simulations [24] (both included in the R2WinBUGS library) was used to assess the quality of the MCMC output. In fact, the Gelman and Rubin statistic has been checked to be lower than 1.1 and the effective sample size to be above 100 for all the parameters saved in the simulation process.

## Results

**Figure 1:**
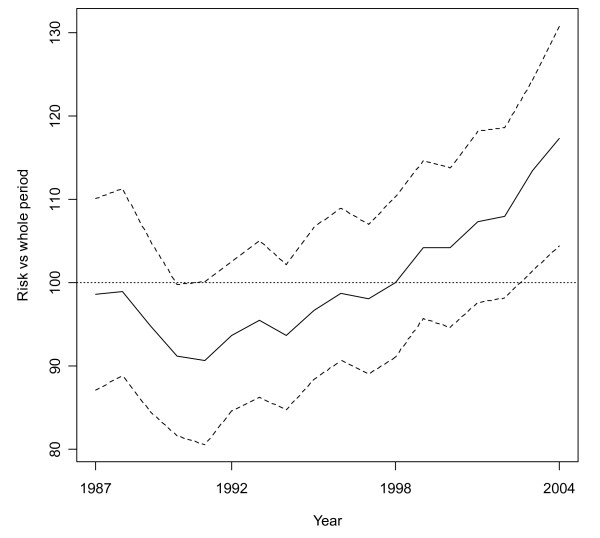
Estimated mean time trend for all the municipalities, period 1987 to 2004. The value 100 indicates the mean risk value for the whole period studied. Dotted lines stand for the 95 percent credibility band.

A total of 2,641 lung cancer deaths in women in the CV were registered from 1987 to 2004. Figure 1 shows the estimated yearly time trend for risk mortality (the parameter Risk Year from the model in the Methods section), the values corresponding to 100 for the risk representing the mean risk level for the whole period. Thus, at the beginning of the period of study, risk decreases until 1991 when it is only 90.66 percent of the risk for the whole period. Nevertheless, from that year until 2004, risk increases constantly and ends up being 17.34 percent higher in the final year compared to the average risk for the whole interval.

**Figure 2:**
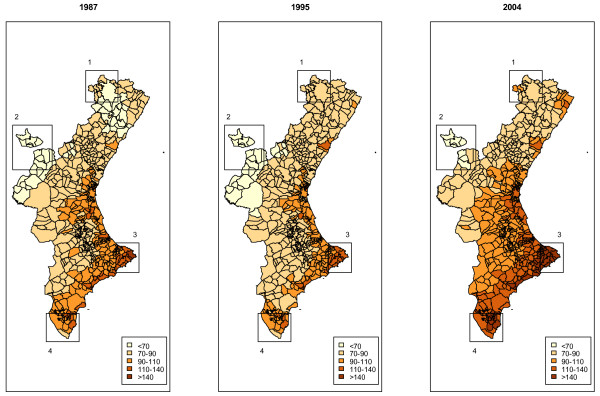
Geographical representation of the spatio-temporally smoothed SMR in every municipality. Years 1987, 1995 and 2004....

Figure 2 shows the estimated smoothed spatio-temporal Standardized Mortality Ratio for the years 1987, 1995 and 2004 in every municipality of the CV. The remaining years are not shown for reasons of space, although they are available as supplementary material [see Additional file 2]. As can be seen in Figure 1, the mean risk estimate for 1987 has a similar value to that in 1995, but both of them are substantially lower than the one obtained in 2004. Therefore, the geographical distribution of risk for 1987 and 1995, in Figure 2, looks similar in terms of darkness of the map, but the map for 2004 is generally darker than those for previous years. Moreover, differences in risk distribution from 1987 to 1995 are minimal. The most relevant changes in this period seem to be a low increase in risk in the north of the CV and a slight decrease in the south, mainly in the area between Zones 3 and 4. However, changes from 1995 and 2004 are greater. Despite the general increase in risk in the whole of the CV, it can be noted that the southern part has experienced a higher increase in risk, Zones 3 and 4 being the ones with the greatest rises.

**Figure 3:**
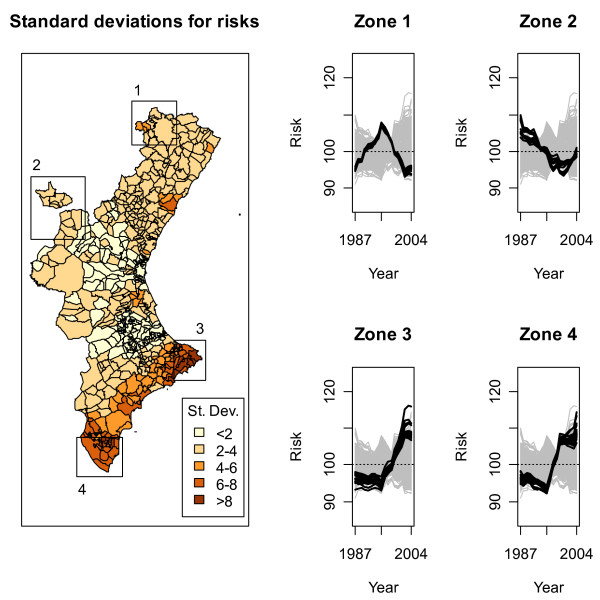
Left side: variability of the estimated smoothed SMR for every municipality. Darker zones point to those regions with higher evolution through the period 1987 to 2004. Right side: Risk time trends for municipalities in zones 1–4 on the left side.

In order to explore the magnitude of the geographical variation in risk during the whole period, on the left of Figure 3 there is a choropleth map with the standard deviations in risks for the 18 years studied for every municipality (more precisely, the standard deviation of 100*exp(SpatioTemporal [j,i]), j = 1, ..., 18 is displayed for every municipality i). This map points towards Zones 3 and 4 as those with the highest variations in risk during the period 1987 to 2004, followed by the region that joins both zones.

On the right hand side of Figure 3 time trends for risk for all the 540 municipalities are shown (represented as grey lines). Each one of the four plots corresponds to one of the highlighted zones on the map, the black lines showing the temporal evolution of risk for the municipalities either partially or totally included in the squares that define the regions. Risks shown are standardized in such a way that a value of 100 stands for the mean value for any municipality for the whole period. Being more precise, in terms of the notation used in the model included as supplementary material, the time trend for municipality *i *shown on the right of Figure 3 corresponds to the following:

$$\begin{array}{cc} RR[j,i]=100\frac{\exp(SpatioTemporal[j,i])}{\exp(mean(SpatioTemporal[•,i]))} & j=1,...,18 \end{array}$$

As can be seen from the former expression, the mean time trend has not been included in Figure 3, but is present in the maps in Figure 2. It can be seen that Zone 1 showed its highest risk in the middle of the period studied, as was suggested from Figure 2. Risk in Zone 2 decreases with time. Zones 3 and 4 have similar patterns: at the start they are more or less flat with very little variation, but both of them experience an abrupt increase from 1995, decreasing in Zone 4 around the year 2000, but staying constant in Zone 3 until the end of the period.

Lastly, the estimated temporal coefficient in every municipality has had a posterior mean value of 0.982 with a 95 percent Bayesian credibility interval (CI) of: [0.945, 0.999]. Further posterior means for standard deviations of the random spatial effects have come out as 0.353 (95 percent CI: [0.222, 0.606]) for the first year and 0.065 (95 percent CI: [0.043, 0.119]) for the increases in consecutive years. Therefore, the need to consider different precision parameters for the first and consecutive periods seems evident.

## Discussion

The present study has shown the different evolution for lung cancer mortality in women in the CV during the period 1987 to 2004. The methodology used has made possible the description of very different time trends in risk without resorting to previously defined shapes, for example linear or quadratic, that would have constrained the way in which time trends are allowed to vary. Moreover, such methodology gathers information from consecutive years to provide robust estimates based on neighbours both in time and space simultaneously. The magnitude obtained for the temporal coefficient (very distant from 0) warns against ignoring this kind of dependence in data.

Unfortunately, there is neither data on smoking for the Spanish population as a whole, nor in the CV, from health interviews for periods prior to the nineties. It has been shown that smoking was rare among Spanish females before the 1958–1962 calendar period through to the period 1968–1972 [25]. Other studies [12] confirm this because the analysis of the smoking prevalence in Europe for 1950–1990 showed that southern European countries, including Greece, Italy, Portugal and Spain, were characterized by low female prevalence rates and sharp gender differences in smoking habits.

The evolution of smoking prevalence among women in the CV can only partially explain the trend described. The data from health interview surveys in the CV [26,27] showed an increase for female smoking prevalence, but this was not too strong: from 22,6 percent in 1991 to 24,5 percent in 2001. However, this increase is partially due to demographic changes in the CV population as, if we considered the same population for year 2001 and for 1991, the new prevalence would only have been 23.8 percent, and therefore the smoking habits of women from the CV do not seem to have undergone significant changes in the period studied. Moreover, the biggest changes in smoking habits have been experienced in the youngest age groups; prevalence has increased from 36.5 percent to 40.3 percent for women aged 25–44 and from 5.6 percent to 13 percent for women aged 45–64. Therefore, the bulk of new women smoking in the CV were very young during the period of study and if it is taken into account that only 26.8 percent of observed lung cancer deaths correspond to women aged below 65, tobacco does not seem to be the only factor responsible for the sharp increase in lung cancer observed.

The geographical distribution clearly shows two zones with higher mortality increases in recent years. The most important demographic characteristic in these two zones is the concentration of foreign population: in 2003 the census [28] showed that in the CV the biggest foreign population was British (14 percent of foreigners), and German nationals were the third (9 percent). Over the decades (from 1970 until the mid nineties) the United Kingdom and Germany have been the main countries of origin for foreigners in the CV (36.2% in 1970, 40% in 1991). Population that migrated from the United Kingdom and Germany have concentrated mainly in the Province of Alicante (on the south of the region) since the seventies: as early as 1981 these two nationalities represented 44.3% of the foreign people in the province and a very high proportion (92%) of British people in the CV were living in that province (76% for German people). In 2001, that proportion was almost the same for British people (91%) and for German people had risen to 88%. Even so, this population was concentrated in specific areas [29]: 80 percent of British people in the CV reside in precisely the two zones with a higher increase in lung cancer mortality in women.

People from these two countries (United Kingdom and Germany) living in the CV are older than the Spanish population or other foreign populations [28]: in 1991 63% of them were 50 years or more (for the total foreign people the proportion aged 50 years or more was 50.6%). Ten years later, the age structure was more or less the same for British and German people living in the CV: 68.8% of them were 50 years or more, meanwhile for all the foreigners the proportion of the population over 50 years was 28.6%.

The age and sex distribution for the British and German population in the CV showed few changes in the nineties, but the variations were in the sense of an increase in female ageing: 60.7% of the female population in 1991 was 50 years and more and ten years.

The smoking situation in the nineties in Europe [30] showed a higher prevalence among British and German women than in Spain, and had been even higher in British women in the previous years. It is possible to suppose that immersed in the international retirement migration within Europe [31] from northern to southern countries a risk import (the female smoking prevalence in northern countries) has taken place in the two CV Zones 3 and 4. The effect of migration on mortality has already been argued [32] to be one of the causes which explains the higher incidence of coronary heart disease in one of these zones. It has been shown that migration, particularly of elderly people, has had an impact on cancer incidence in Florida [33], the North American state with a great migration flow (retirement and Caribbean migration), where 15 counties have more than 20 percent of their population over 65 years old.

**Figure 4:**
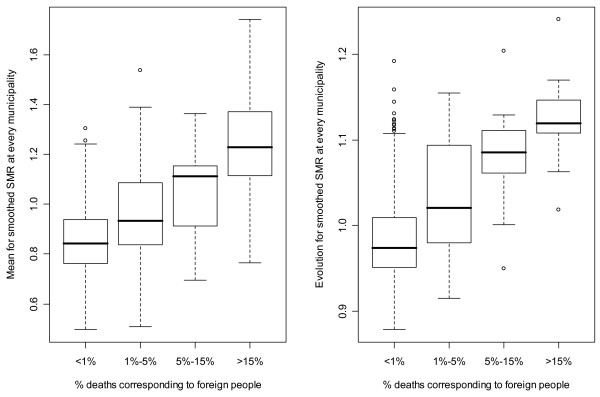
Left side: distribution of the mean risk as a function of the percentage of foreign deaths for every municipality. Right side: distribution of the relative risk from 1987 to 2004 as a function of the percentage of foreign deaths for every municipality.

In order to relate migration and the results shown in the previous section, Figure 4 shows the relation between the smoothed SMR resulting from the model in Appendix A and the percentage of deaths corresponding to foreign population for every municipality in the CV during the period 1991–2000 (It has not been possible for us to obtain such covariate for the whole period 1987–2004). The percentage of deaths corresponding to foreign population in municipalities of the CV ranges from 0% to 59.71% and this variable has been split into 4 categories: <1% of deaths corresponding to foreign population (400 municipalities), 1%–5% of deaths corresponding to foreign population (85 municipalities), 5%–15% (29 municipalities), >15% (26 municipalities). On the left side of Figure 4 is shown a boxplot relating the percentage of foreign deaths for every municipality with its mean value of the risk for the whole period excluding the time trend for the whole CV (in terms of the model in Appendix A for the i-th municipality it would correspond to exp(mean(SpatioTemporal [.,i]))). Therefore, it can be seen that those municipalities in which the foreign population has a substantial contribution to the total number of deaths are mainly those municipalities with higher risk of lung cancer mortality in women. Statistical significance has been assessed for this association (P-value of 2.2e-16 for ANOVA). At the right side of Figure 4 the boxplot relates the percentage of foreign deaths with the increment in the smoothed SMR from period 1 to 18 for every municipality (in terms of the model in Appendix A for the i-th municipality it would correspond to exp(SpatioTemporal [18,i]- SpatioTemporal [1,i])). It can be noticed again that those municipalities with a higher percentage of foreign deaths are mainly those with a higher increase in its smoothed SMR. In this case statistical significance has also been assessed for this relation (P-value of 0 for ANOVA).

As a limitation of the present work, we would like to point out that the association between migration and lung cancer mortality in women has been established at an ecological level and an individual study would be very useful to confirm the hypothesis arising from this work. Moreover, we would like to draw attention to the difficulties associated with including covariates in our work, as it is well known that lung cancer is a disease with a long latency, thus the occurrence of the disease can be the consequence of the exposure to a risk factor during a long time, about 30 years [7]. Therefore the covariates to include in the model should be related to a much earlier moment in time but we don't know which one with any precision. Moreover, some covariates of interest for our problem are not available with such a long latency time. Due to all these difficulties it hasn't been possible to include the covariates in the proposed model and it has been necessary to relate the smoothed SMR to an indirect variable, being the percentage of foreign deaths at every municipality.

On the other hand, an ecological bias may exist: were the women who died from lung cancer Spaniards or foreigners? Against this bias is the high proportion of foreigners in the municipalities affected (higher than 30 percent of the total population) [28]. Moreover, the region between Zones 2 and 3 (the surroundings and metropolitan areas of the cities of Alicante and Elx) have a lower female lung cancer mortality increase and are inhabited mainly by Spanish people. These are the two coastal municipalities in the south of the CV that have been least affected by residential migration. In addition, there are other Spanish regions with an elevated proportion of foreigners from retirement migration like Malaga (*"Costa del Sol"*) where the female lung cancer mortality rate has also undergone a large increase [34], or the Canary Islands with a rate ratio against Spain for female lung cancer higher than 1.5 for the period 1978 to1992 [35]. Nevertheless, a specific mortality study for female foreigners is needed to ensure the avoidance of the effect of an ecological bias.

If the hypothesis of risk importation is confirmed, there will be important consequences for prevention which should be taken into account in those regions with significant elderly migration (Algarve, Portugal; Tuscany, Italy; Costa del Sol, Spain; Florida, United States...). Primary prevention will be difficult as the duration of smoking has been long for women in their native countries and they are probably middle aged and elderly women for whom the effectiveness of counselling to give up tobacco will be limited. This implies that efforts will have to be aimed mainly towards secondary prevention and treatment, with more expensive consequences both in economic terms and life-loss terms.

## Conclusion

Spatio-temporal evolution of lung cancer mortality in women for the period 1987–2004 has not been the same for all the regions in the CV. The southern coast of this region has experienced a higher increase in risk during the observed period. Migration should be considered when studying lung cancer mortality trends, mainly in tourist regions, as people coming from high risk factor countries can modify mortality behaviour in the host country. This fact can have important consequences for lung cancer prevention. The temporal term in disease mapping provides a closer approach to risk factors acting on the population and this term can be appropriately described with a suitable Bayesian hierarchical model.

## Competing interests

The author(s) declare that they have no competing interests.

## Authors' contributions

OZ: He has been the main director of the study and has developed all the epidemiological aspects of the work.

HV: Jointly with Dr. Zurriaga, she has been in charge of all the epidemiological aspects of the study and has collaborated intensely in the discussion of the results.

MAM: He has provided and supervised the methodology used in the paper.

PB: She has taken on the data analysis, run the models and performed the complementary statistical analyses in the discussion section.

## Pre-publication history

The pre-publication history for this paper can be accessed here:



## Supplementary Material

Additional File 1 Winbugs syntax for the model used in the analysis. Winbugs syntax corresponding to the model described in the methods section. Click here for file

Additional File 2 Spatio-temporal risk smoothing for the years 1987 to 2004. Annual maps for the years from 1987 to 2004 showing the spatio-temporal estimates for the SMR in every municipality of CV. Click here for file

## References

[B1] Levi F, Lucchini F, Negri E, La Vecchia C (2004). Trends in mortality from major cancers in the European Union, including acceding countries, in 2004. Cancer.

[B2] Bray F, Tyczynski JE, Parkin DM (2004). Going up or coming down? The changing phases of the lung cancer epidemic from 1967 to 1999 in the 15 European Union countries. Eur J Cancer.

[B3] Levi F, Lucchini F, Negri E, La Vecchia C (2007). Continuing declines in cancer mortality in the European Union. Ann Oncol.

[B4] Sánchez-Hernández I, Izquierdo-Alonso JL, Almonacid-Sánchez C (2006). Epidemiology of Lung Cancer in Spain and Forecast for the Future. Arch Bronconeumo.

[B5] Bosetti C, Levi F, Lucchini F, Negri E, La Vecchia C (2005). Lung cancer mortality in European women: recent trends and perspectives. Ann Oncol.

[B6] Bernal Pérez M (2002). Increase of lung cancer in women (in Spanish). Med Clin (Barc).

[B7] Weiss W (1997). Cigarette Smoking and Lung Cancer Trends. A light at the end of the tunnel?. Chest.

[B8] Tyczynski JE, Bray F, Parkin DM (2003). Lung cancer in Europe in 2000: epidemiology, prevention, and early detection. Lancet Oncol.

[B9] Lopez-Abente G, Pollán M, Aragonés N, Pérez B, Llácer A, Pérez J (2002). Mortality trends in Spain, 1952–1996: age, cohort and period effect (in Spanish).

[B10] European Commission (2003). The health status of the European Union Narrowing the health gap.

[B11] Molarius A, Parsons RW, Dobson AJ, Evans A, Fortmann SP, Jamrozik K, Kuulasmaa K, Moltchanov V, Sans S, Tuomilehto J, Puska P (2001). Trends in cigarette smoking in 36 populations from the early 1980s to the mid-1990s: findings from the WHO MONICA project. Am J Public Health.

[B12] Graham H (1996). Smoking prevalence among women in the European community 1950–1990. Soc Sci Med.

[B13] Brennan P, Bray I (2002). Recent trends and future directions for lung cancer mortality in Europe. Br J Cancer.

[B14] Martínez Beneito MA, López Quílez A, Amador Iscla A, Melchor Alós I, Botella Rocamora P, Abellán Andres C, Abellán Andrés JJ, Verdejo Mañez F, Zurriaga Lloréns O, Vanaclocha Luna H, Escolano Puig M (2005). Mortality Atlas of the Comunitat Valenciana 1991–2000 (in Spanish) Valencia (Spain).

[B15] Pickle LW, Mungiole M, Jones GK, White AA (1999). Exploring spatial patterns of mortality: the new atlas of United States mortality. Stat Med.

[B16] Devesa SS, Grauman DJ, Blot WJ, Fraumeni JF (1999). Cancer surveillance series: changing geographic patterns of lung cancer mortality in the United States, 1950 through 1994. J Natl Can Inst.

[B17] (1979). International Classification of Diseases, 9th revision, Clinical Modification.

[B18] World Health Organization (1992). International Classification of Diseases, (ICD-10) Tenth Revision, WHO edition.

[B19] Martínez-Beneito MA, López Quílez A, Botella Rocamora P (2007). An autoregressive approach to spatio-temporal disease mapping. Statistics in medicine.

[B20] Spiegelhalter DJ, Best NG, Carlin BP (2002). Bayesian measures of model complexity and fit (with discussion). Journal of the Royal Statistical Society: Series B (Statistical Methodology).

[B21] Spiegelhalter DJ, Thomas A, Best N, Lunn D (2002). WinBUGS User Manual, Version 14.

[B22] R Development Core Team (2006). R: a language and environment for statistical computing.

[B23] Brooks SP, Gelman A (1998). Alternative methods for monitoring convergence of iterative simulations. Journal of computational and graphical statistics.

[B24] Gelman A, Carlin JB, Stern HS (2004). Bayesian data analysis.

[B25] Schiaffino A, Fernández E, Borrell C, Salto E, García M, Borrás JM (2003). Gender and educational differences in smoking initiation rates in Spain from 1948 to 1992. Eur J Public Health.

[B26] Conselleria de Sanitat i Consum (1993). Health survey of the Comunitat Valenciana 1990–1991 (in Spanish).

[B27] Conselleria de Sanitat (2001). Health Plan of the Comunidad Valenciana 2001–2004 (in Spanish).

[B28] Perfiles de los extranjeros residentes en la Comunidad Valenciana: nacionalidad, edad y sexo (in Spanish). Observatorio Valenciano de las Migraciones. Colección Miradas sobre la Inmigración. Núm. 1. http://www.ceimigra.net/viejaweb/ceim_home/miradas/miradas01.pdf.

[B29] La distribución territorial de la población extranjera en la Comunidad Valenciana (in Spanish). Observatorio Valenciano de las Migraciones. Colección Miradas sobre la Inmigración. Núm. 13. http://www.ceimigra.net/viejaweb/ceim_home/miradas/miradas13.pdf.

[B30] World Health Organization (WHO). Prevalence of smoking by country, women aged 15 and over, 1980–2000 Europe. WHO. European Health for All Database. http://www.euro.who.int/hfadb.

[B31] King R, Warnes AM, Williams AM (1998). The international retirement migration in Europe. Int J Popul Geogr.

[B32] Librero Lopez J, García Benavides F, Godoy Laserna C (1993). An analysis of mortality in small areas: the problem of residency (in Spanish). Gac Sanit.

[B33] McCoy HV, Ritchey PN, McCoy CB (1992). Effects of migration on cancer incidence and resources for prevention and treatment in Florida. Public Health Rep.

[B34] Cayuela A, Rodríguez-Domínguez S, Otero R (2006). Trends in lung cancer mortality rates in the provinces of Andalusia, Spain, 1975–2002 (in Spanish). Arch Bronconeumol.

[B35] López-Abente G, Pollan M, Escolar A, Errézola M, Abraira V (1996). Atlas of cancer mortality and other causes of death in Spain 1978–1992.

