# Supplementary material for: Spatio-temporal evolution of female lung cancer mortality in a region of Spain, is it worth taking migration into account?
Source: BMC Cancer. 2008 Jan 31;8:35. doi: 10.1186/1471-2407-8-35 (PMC2267803; doi:10.1186/1471-2407-8-35)

SUPPLEMENTARY MATERIAL TO INCLUDE IN THE WEB PAGE

MAPS FOR THE SPATIO-TEMPORALLY ESTIMATED SMR

1987

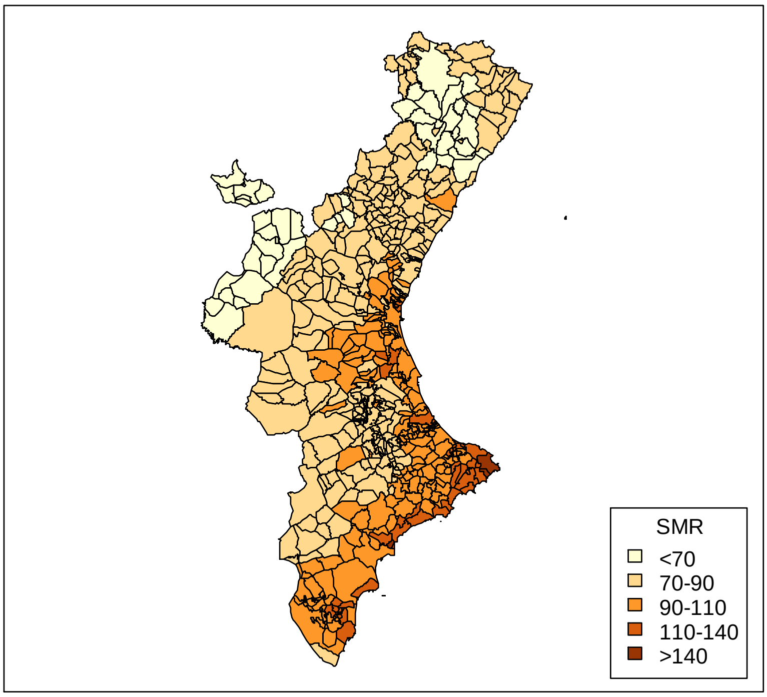

1988

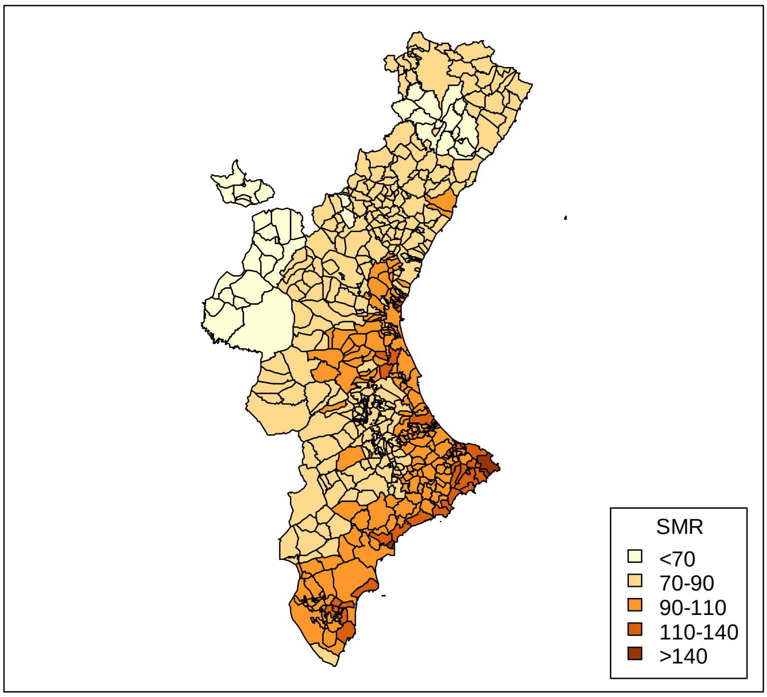

1989

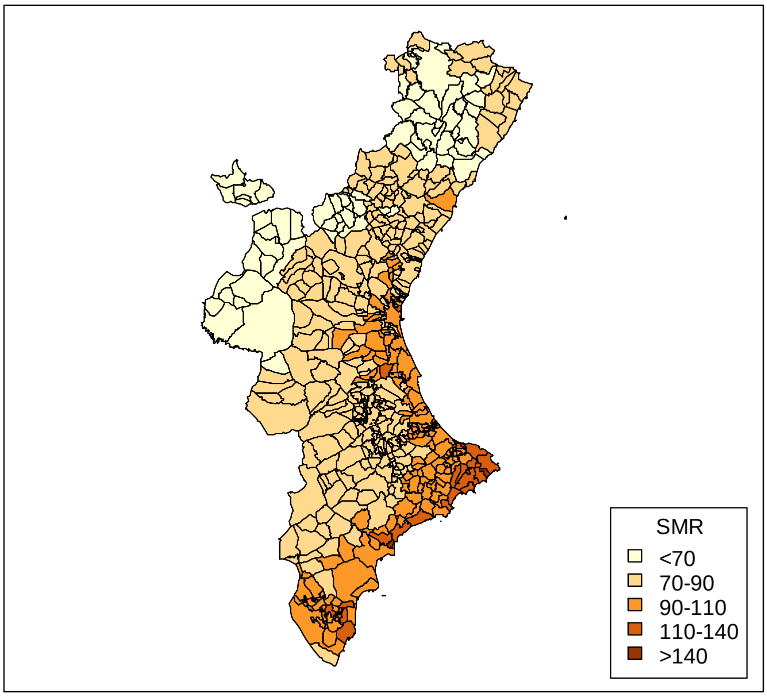

1990

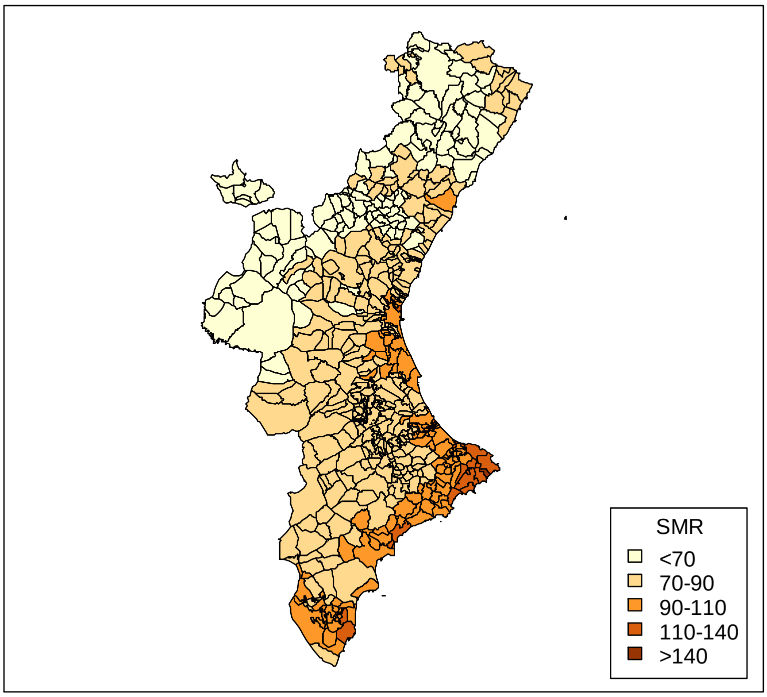

1991

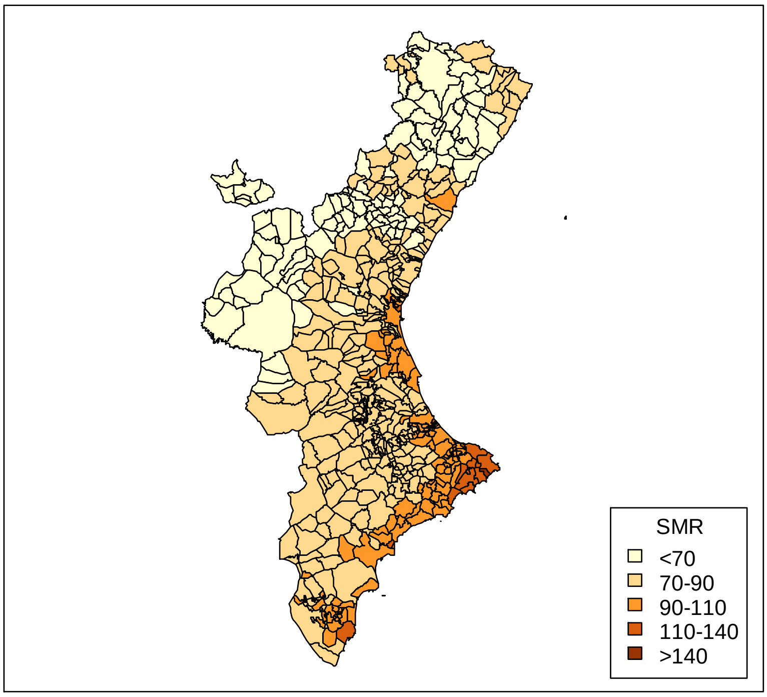

1992

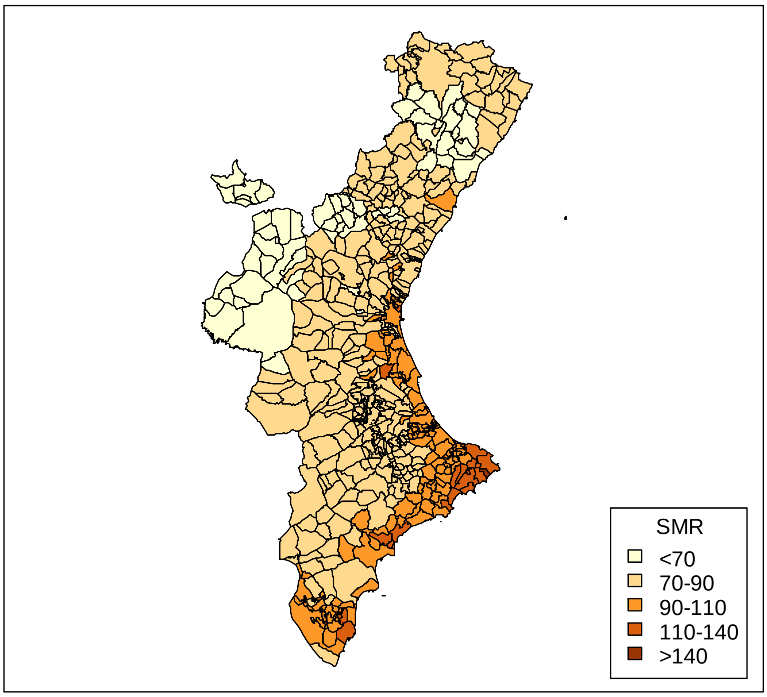

1993

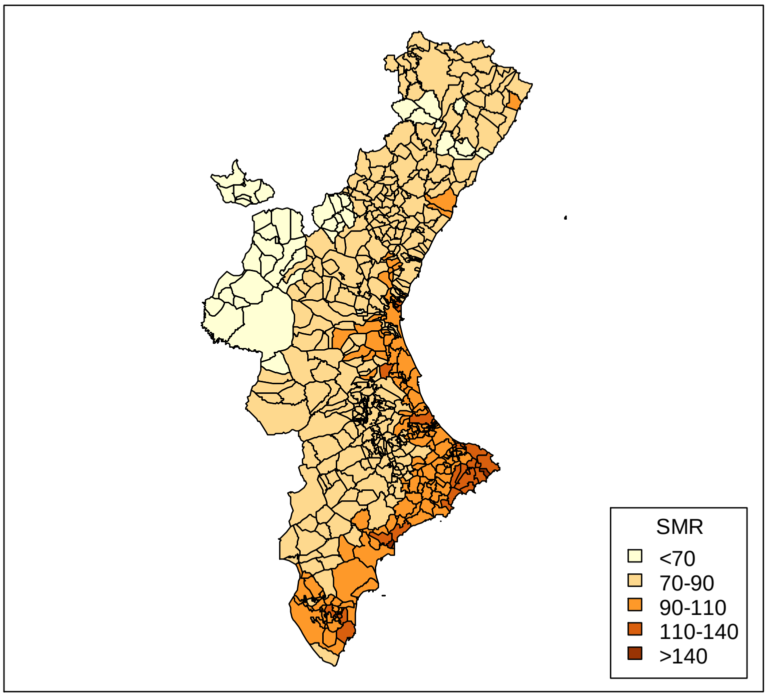

1994

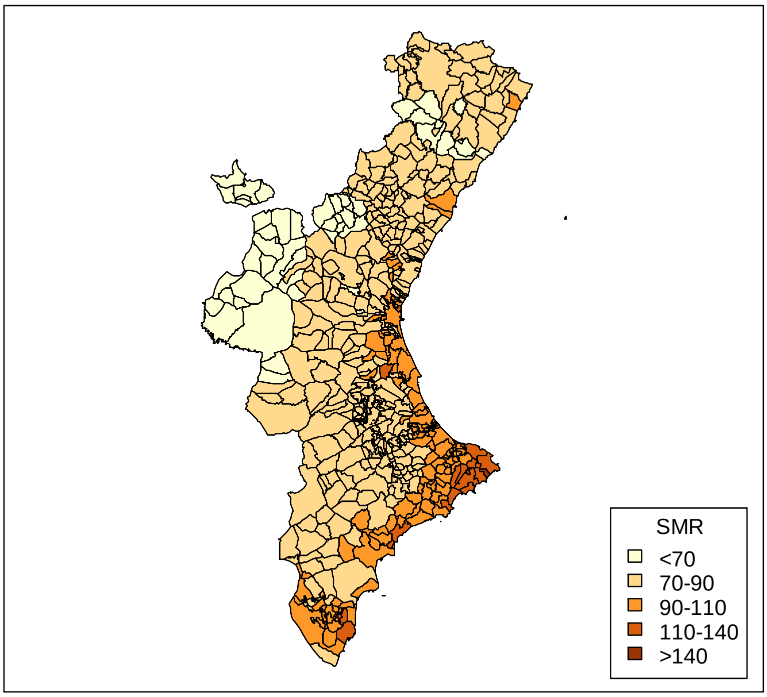

1995

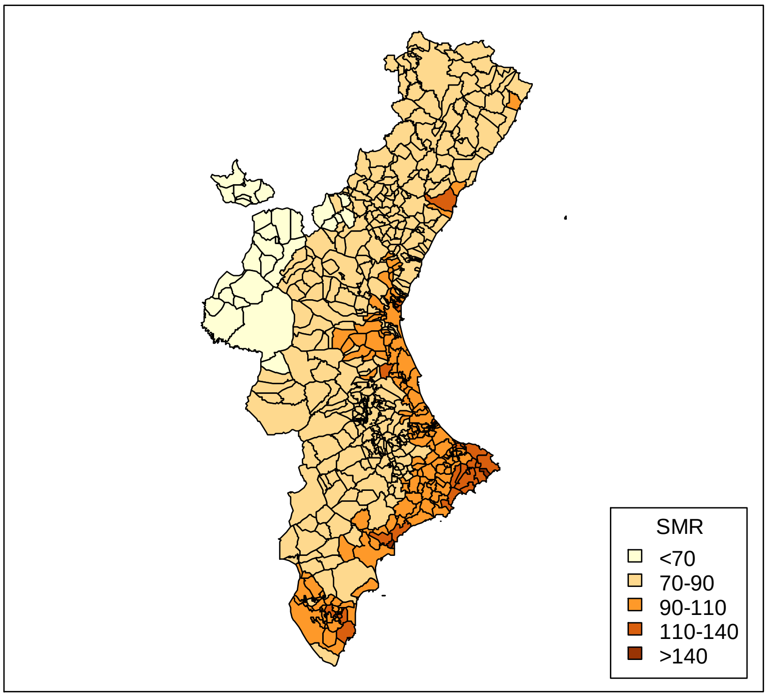

1996

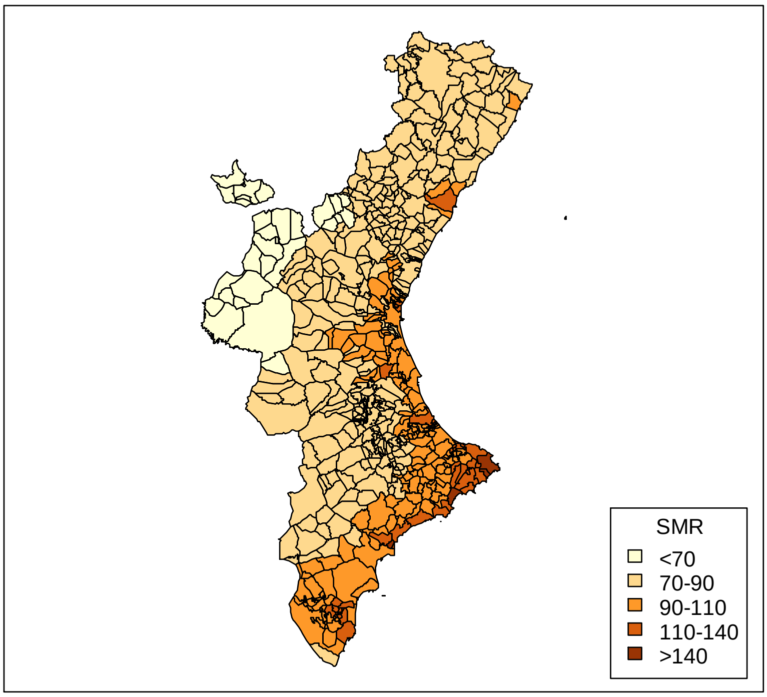

1997

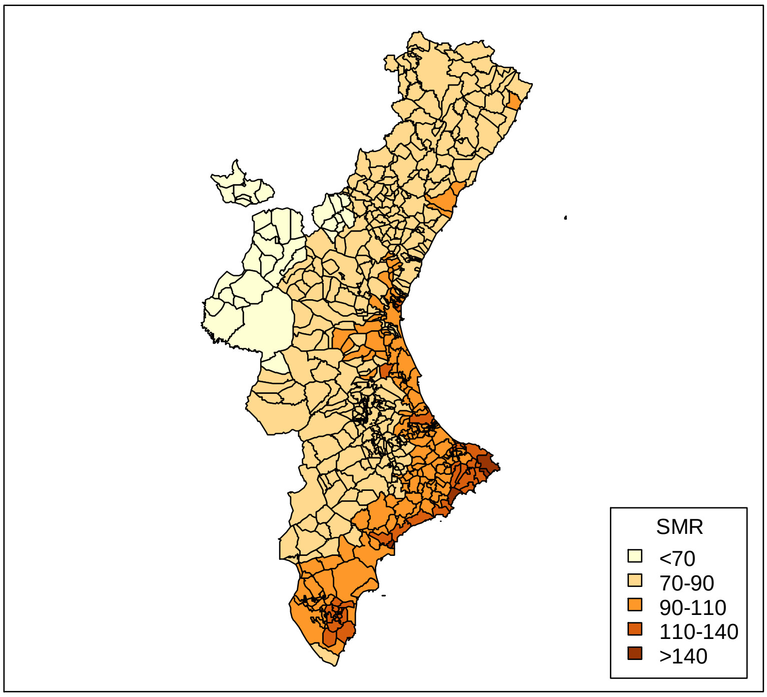

1998

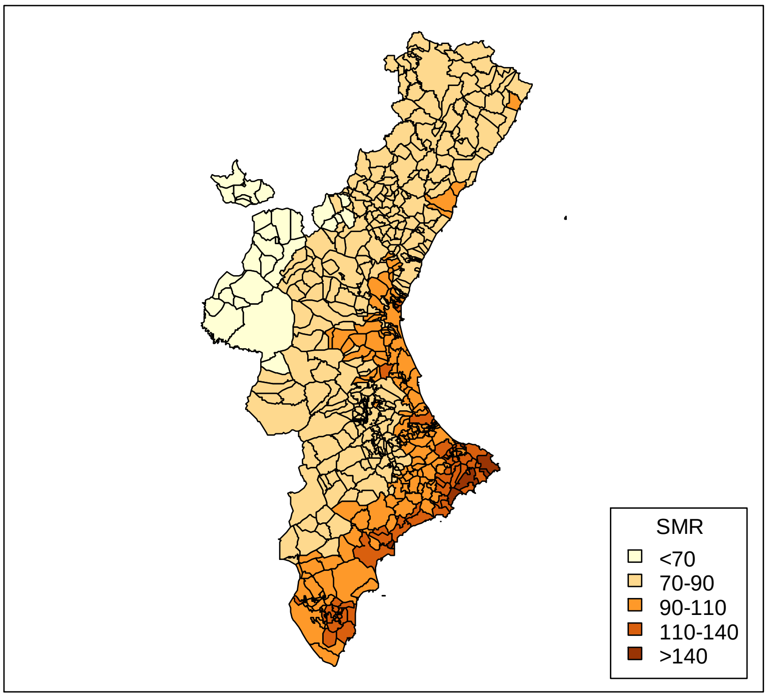

1999

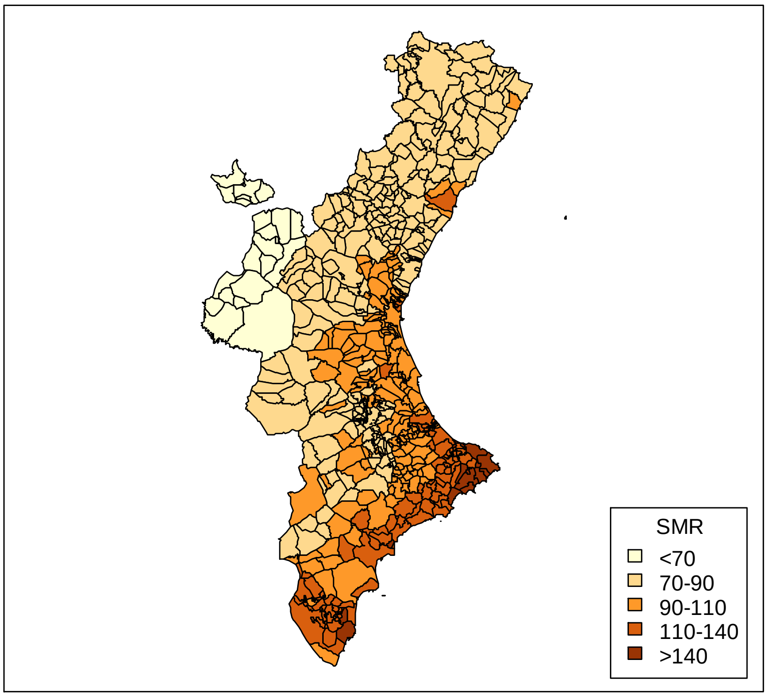

2000

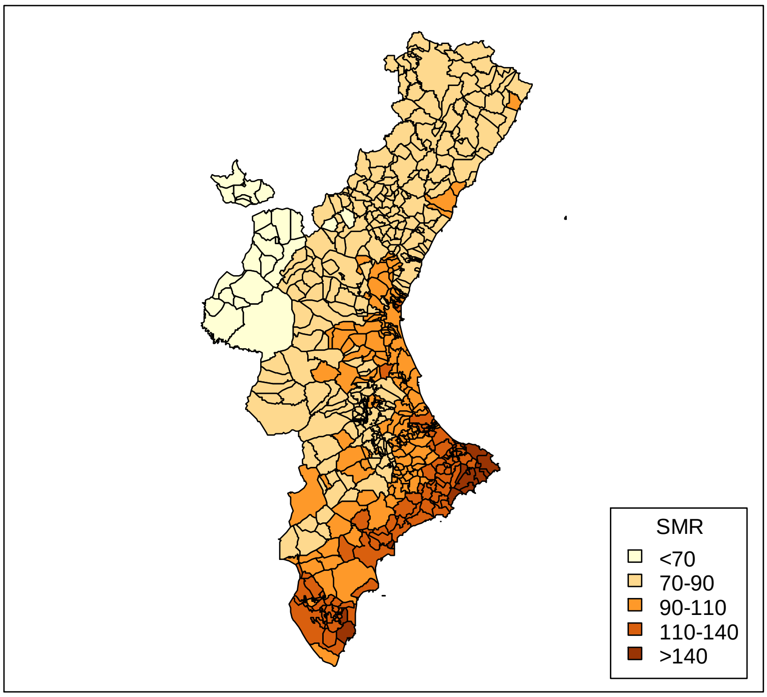

2001

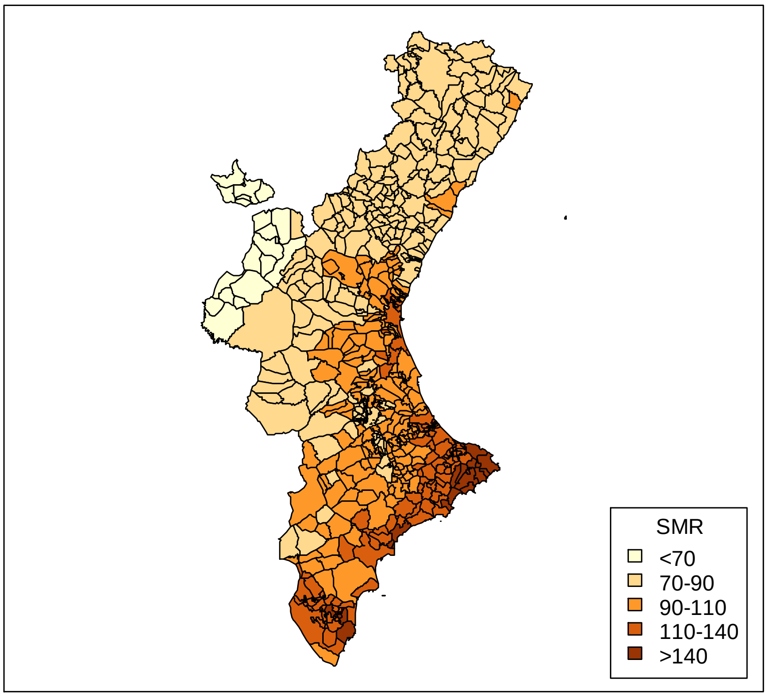

2002

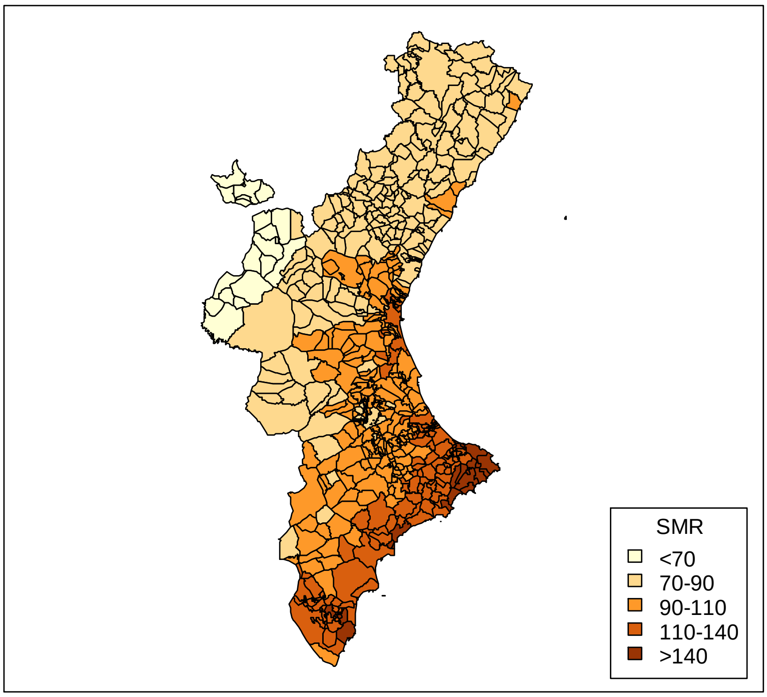

2003

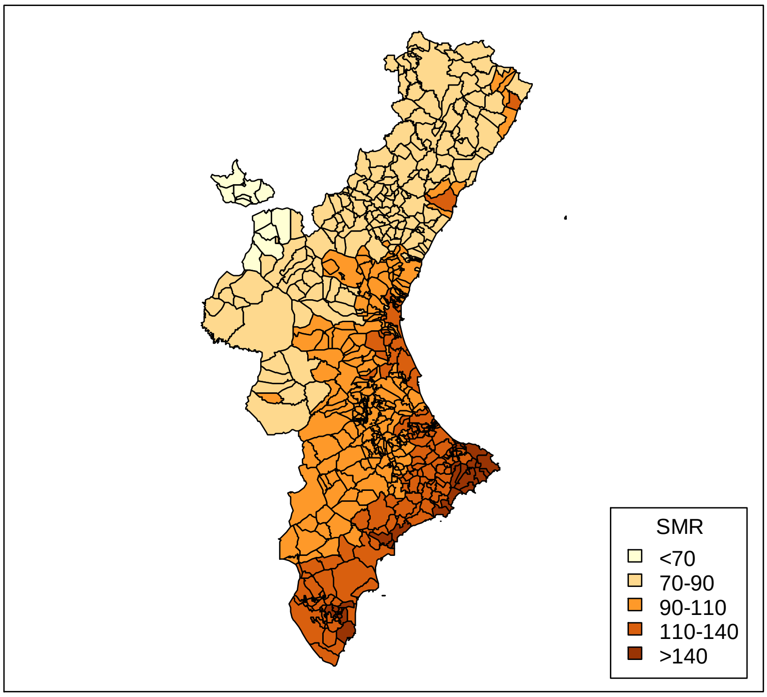

2004

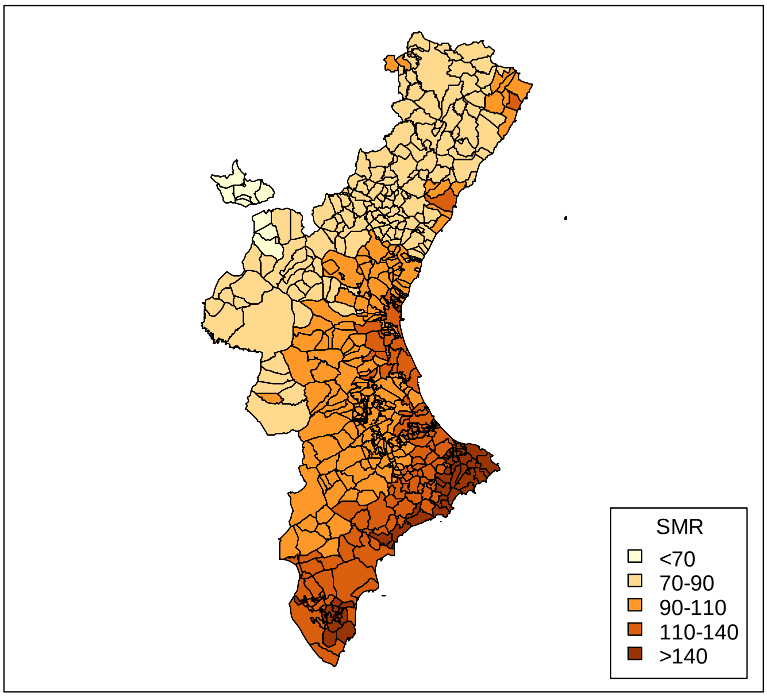

Supplement: Additional File 2 — Spatio-temporal risk smoothing for the years 1987 to 2004. Annual maps for the years from 1987 to 2004 showing the spatio-temporal estimates for the SMR in every municipality of CV. [file 1471-2407-8-35-S2.pdf]
